# How article category in Wikipedia determines the heterogeneity of its editors

**DOI:** 10.1038/s41598-023-50448-y

**Published:** 2024-01-07

**Authors:** Aileen Oeberst, Till Ridderbecks

**Affiliations:** 1 Department of Psychology, University of Hagen, 58084 Hagen, Germany; 2 Leibniz Institut für Wissensmedien, Wissenskonstruktion, Tübingen, Germany

**Keywords:** Psychology, Human behaviour

## Abstract

Collaboration is essential to advancing knowledge and, ultimately, entire societies. With the development of Web 2.0, the possibilities have risen to unprecedented levels and allowed for the collaborative creation of the world’s largest compendium of knowledge that ever existed – Wikipedia. Collaboration is not a safeguard of quality per se, however. Rather, the quality of Wikipedia articles rises with the number of editors per article as well as a greater diversity among them. Here, we address a not yet documented potential threat to those preconditions: self-selection of Wikipedia editors to articles. Specifically, we expected articles with a clear-cut link to a specific country (e.g., about its highest mountain, “national” article category) to attract a larger proportion of editors of that nationality when compared to articles without any specific link to that country (e.g., “gravity”, “universal” article category), whereas articles with a link to several countries (e.g., “United Nations”, “international” article category) should fall in between. Across several language versions, hundreds of different articles, and hundreds of thousands of editors, we find the expected effect within Wikipedia: The more exclusively an article topic is linked to a particular nation, the higher the proportion of editors from that country is among the contributors.

## Introduction

Collaboration and cooperation are of great importance for societies and their development^1,2^. After all, building on the ideas of others is essential to advancing knowledge and, ultimately, entire societies. With the development of Web 2.0, the possibilities to collaborate have risen to unprecedented levels and enabled harnessing the “wisdom of the crowd”^3^. This resulted–among other things–in solving hitherto unsolved mathematical problems^4^ as well as the creation of the world’s largest compendium of knowledge that ever existed – Wikipedia (https://en.wikipedia.org/wiki/Wikipedia:Size_in_volumes). However, collaboration is not better per se^5^. Groups and even crowds may succumb to the same biases as individuals^6–10^, and under certain circumstances, collaboration can even have detrimental effects: When like-minded people work together, they may become more extreme (*group polarization*^11–14^) and they may show even more bias than individuals^15^. This is likely the case because people sharing a perspective (i.e., opinion, bias) tend to overlook relevant information^16,17^. Groups comprising diverse perspectives, in contrast, more likely consider information that is inconsistent with their prior beliefs^18^, which is a safeguard against biases^19^ and, thus, reduces one-sided information processing^17,20^. In line with this reasoning, the quality of Wikipedia articles, for instance, has been linked to the numbers of editors^21,22^ as well as the diversity among them^9,22–24^.

Although diversity may have clear benefits for collaboration, it is rather difficult to achieve: In contrast to the lab, where participants are often randomly assigned to conditions and potential collaborators, people in the real world are often free to choose with whom and for what they want to collaborate. People may choose, for instance, whether or not they want to actively contribute to Wikipedia or not. Or whether they prefer to contribute to another online-encyclopedia, such as Conservapedia, instead. In research, this process is denoted as *self-selection* (i.e., people choose among a variety of opportunities and select themselves to stimuli, environments, or people) and typically, it is regarded as a potential source of bias (*self-selection bias*^25^). After all, it is *certain* people who self-select to *certain* conditions. For instance, people who are attracted to psychological studies and take part in them have more symptoms of personality disorders^26^. The results of the famous Stanford Prison Experiment even might have been (partly) the consequence of self-selection due to the fact that *certain* people are attracted to take part in a study on “prison life”, namely those who score higher on aggressiveness, authoritarianism, narcissism, social dominance and who score lower on empathy and altruism^27^. In the same vein, certain people choose to join police forces^28^ and people self-select to schools that match their values^29^. Taken together, self-selection typically takes place along shared characteristics of the selves. This may have beneficial consequences. For instance, it may foster cooperation among those who self-selected to cooperative contexts^30,31^. But it may also have detrimental consequences – even in the context of cooperation: As people are rather drawn towards like-minded people (*social homophily*^32,33^) and, thus, tend to self-select to contexts where they expect to encounter others that share their views^34–38^, rather homogeneous groups and networks emerge^13,39^, in which people are mainly exposed to opinions that match their own (*echo chambers*^40,41^). Gillani and colleagues^42^ even speak about a trend towards *“ideological cocooning”* on social media platforms (but see^43,44^). Even for online encyclopedias that strive for the representation of generally accepted *knowledge*, self-selection effects have been obtained: As field and lab research by Krebs and colleagues^5^ shows, people prefer to contribute to online encyclopedias /Wikis that match their own attitudes. For instance, in a field study they compared articles between the three online encyclopedias Wikipedia, Conservapedia and RationalWiki (which has been founded as a counterpart to Conservapedia) and found that the most prolific editors in Conservapedia were significantly more conservative than those in Wikipedia and RationalWiki, whereas the most prolific editors in RationalWiki were more liberal than those in Wikipedia and Conservapedia. More importantly, when comparing articles about the same topics (e.g., Abortion, Death Penalty) between the three online encyclopedias as well as the expert-written encyclopedia Britannica, Conservapedia articles and RationalWiki articles deviated significantly from Britannica in that they were more conservative and more liberal, respectively. In other words, self-selection of editors translated into biased articles. Only Wikipedia articles were comparable to Britannica articles in terms of a balanced representation of the topic (see also^45^).

But also Wikipedia is not free from bias, however: As predominantly men self-select to Wikipedia^46^, it might be of little surprise, that a gender bias has been obtained^47,48^. Similarly, as Wikipedia editors are dominated by Western contributors^49,50^, Wikipedia is culturally biased towards Western perspectives. Consequently, self-selection, again, translates to imbalances in the authorship, which in turn, translate into imbalances in content. But self-selection does not end with the decision to contribute to Wikipedia (or other collaborative projects). Rather, editors of Wikipedia further self-select to tasks: They decide, which articles to create, which to ignore, which to edit and what to edit. Consequently, self-selection could, again, produce unequal distributions and might, thus, contribute to bias at this level. To the best of our knowledge, the present paper is the first to investigate such self-selection *within* Wikipedia by systematically analyzing and comparing editor composition for certain article categories within Wikipedia. Specifically, we show that the proportion of editors from a certain nationality varies substantially as a function of the article’s link to the editor’s nationality. That is, articles with a clear-cut link to the specific country (e.g., articles about the capital, the prime minister, its highest mountain, etc.) attract a much larger proportion of editors of that nationality when compared to articles without any specific link to that country (e.g., articles about universal topics such as “gravity”, “music”). Articles with a link to several different countries (e.g., articles about the “United Nations”, bilateral political relationships or conflicts, wars, and treaties between nations), then again, comprise the intermediate category.

Why would one expect such a pattern? First, people are ethnocentric and give precedence to the group they belong to^51^. Second, people develop interests for things they got in contact with^52^ and, thus, are more likely interested in topics of their own environment. For instance, people prefer music that is linked to their own group^53^ and films with actors of their own group^54^. Third, topics with a clear link to individuals’ own country are often of greater relevance to their own life (e.g., politicians, historical conflicts, nearby cities, etc.), which furthermore fosters interest in those topics, as does prior knowledge, that is likewise usually more prevalent for topics from one’s environment^55^. Last but not least, school education (e.g., regarding history) but also news media typically have a national focus^56–60^ and, thus, provide more information on topics that are concerning people’s own country compared to topics that are concerning others’ nations. Consequently, one could expect “national” article topics (e.g., about the capital, prime minister, geographical sites) to attract a particularly large proportion of editors from that country – particularly when compared to articles about universal topics without any national link. And as international topics (e.g., about international organizations, international conflicts and international agreements), concern several different countries, these articles should increasingly attract editors who come from the different countries that are concerned – thereby resulting in an intermediate proportion of members of one specific country. For instance, the proportion of Austrian editors should be highest for articles about topics that directly concern Austria, and lowest for articles about universal topics without any direct link to Austria with articles about international topics that concern Austria among other countries in between (i.e., national > international > universal).

## Methods

We tested our hypothesis in three different samples in order to ensure both the internal and external validity of our findings. Table 1 summarizes the data our analyses are based upon.

**Table 1:** Overview about the data.

	Sample 1	Sample 2	Sample 3
Number of language versions of Wikipedia included	1	7	7
Number of articles included	82	567	522
Number of editors analyzed	45,491	10,021	178,947
Number of editors categorized	33,592	6216	178,947
Number of editors categorized unambiguously	12,008	3705	0

### Countries of interest

In order to be able to test our hypothesis, we had to define countries of interest in order to identify article topics that comprise a direct link to this country of interest (i.e., national and international articles, see article selection). Note, that we define countries as sovereign states. As previous general user statistics have shown, there is often a nationality that is predominant among the editors of a language version (https://stats.wikimedia.org/wikimedia/squids/SquidReportPageEditsPerLanguageBreakdown.htm). For instance, the German and Portuguese language versions of Wikipedia are predominantly (>80%) edited by German and Brazilian editors, respectively. Note that these percentages regarded the entire language version of Wikipedia and were not topic-sensitive. As our hypothesis predicted an *increased* proportion of editors of the country of interest for international and national articles, we decidedly selected countries of interest that were *not* generally predominant among the editors in order to avoid ceiling effects, but, of course, had the respective language as official language (e.g., Austria for the German language version with a global share of 7% among the editors; Canada for the French language version with a global share of 4.5% among the editors). After all, if Germans already accounted for more than 80% of all those who *generally* contributed to the German language version of Wikipedia, *increases* in this proportion for international as well as national article topics might be difficult to obtain due to ceiling effects. To avoid this statistical limitation, we opted for countries with generally lower proportions of editors.

### Article selection

We investigated the influence of article topic on the proportion of editors from the country of interest by preselecting Wikipedia articles and assigning them to three different topic categories: universal, international and national. *Universal article topics* were defined as being of universal concern and, thus, lacking a direct link to any particular country. To this end, we created a list of 28 article topics (e.g., about “biology”, “gravity”, “music”), for which articles in all corresponding language versions were selected accordingly (see https://osf.io/sqan3/). *International article topics* were defined as articles about topics that directly concern at least two countries, with one of them being the country of interest in our sample. We selected five different types of international topics and present examples from our Sample 1, where Austria was the country of interest: (1) inter-group conflicts, (2) wars/battles, (3) political or economic agreements, (4) international organizations, (5) international political relationships. For each country of interest, we selected the corresponding articles in the relevant language version of Wikipedia (e.g., the article about the United Nations in the German language version of Wikipedia for the analysis of the proportion of Austrian editors in Sample 1, but also the article about the United Nations in the French language version of Wikipedia for the analysis of the proportion of Canadian editors in Sample 2; see https://osf.io/sqan3/). *National article topics* were defined as exclusively concerning the country of interest. We defined a preset list of topics about geographical sites (e.g., cities, mountains) that are solely and undisputedly located in the country of interest, politicians, celebrities, and national holidays (see https://osf.io/sqan3/). Analogously to the international topics, we selected for each country of interest the corresponding articles from the relevant language version of Wikipedia. For instance, for our analysis of Austrian editors, we selected the article about „Sebastian Kurz“, as he was the Chancellor of Austria at the time of data retrieval. In individual cases, articles were retroactively excluded, because they did not meet all criteria. For instance, although “Elizabeth II” was the head of the state of Canada at the time of data retrieval, the article cannot be considered a “national topic” for Canada as she was also head of the state of other countries. Cases, in which no articles for the predefined topics existed, were treated as missing data.

### Editor identification

We made use of https://xtools.wmflabs.org/articleinfo to extract information about editors that contributed to the selected articles. To ensure a comparability of our findings, we set February 28th of 2019 as an end date. In other words, all samples alike comprise the editors that contributed to the respective articles until February 28th, 2019. Bots were excluded from all analyses. The three samples differ with regard to their coverage and completion: *Sample 1* was limited to one language version (German; country of interest: Austria) but covered the total sample of editors that had contributed to the articles we analyzed. *Sample 2* was extended to seven language versions with corresponding countries of interest (German: Austria, English: Australia, French: Canada, Dutch: Belgium, Portuguese: Portugal, Spanish: Bolivia, Russian: Belarus), but was limited to the *Top Editors* of each article. These are the twenty most prolific editors (i.t.o. number of edits) of each article as identified by xtools. *Sample 3* consisted of seven language versions with corresponding country of interest (English: Australia, French: Canada, Dutch: Belgium, Portuguese: Portugal, Spanish: Bolivia, Russian: Belarus, Arabic: Lybia) but was limited to anonymous editors (i.e., IP addresses) of each article.

### Coding editor nationality

Information on the origin of Wikipedia editors was obtained by two different strategies: For anonymous editors, only IP-addresses were available. These were geo-tracked automatically by an application that was programmed for this purpose (see https://osf.io/sqan3/) and we collected that information at the national level (i.e., the country in which the connected device is located). This type of data was defined as ambiguous as it does not provide clear-cut information with regard to the editors’ nationality. For registered users, we content-analyzed their user pages and had human raters search for information about their nationality (partly with the help of automatic translation tools). To this end, we developed a comprehensive coding scheme (see https://osf.io/sqan3/) and, again, distinguished between ambiguous and unambiguous (i.e., clear) information. As unambiguous we considered information that explicitly conveyed the origin of editors (e.g., by using statements such as “I come from [country]” or by using user boxes in Wikipedia). In contrast, editors’ native language, other language skills or information regarding his or her location (e.g., by mentioning a school or university that was attended, or a work place), were only regarded as ambiguous information regarding the editors’ own nationality. Consequently, we were able to (a) restrict our analyses to editors we could unambiguously categorize or (b) extend our analyses to all editors for whom we had at least found ambiguous information regarding their nationality. We report on both. To determine the reliability of the human coding process, we had a second rater code a subsample of *N* = 1,573 editors. Inter-rater agreement was generally high (Cohen’s Kappa > .80; see Supplemental_Material for more details) and, thus, indicated almost perfect agreement^61^ (for more details see Supplemental_Material).

## Results

### Sample 1—German language version, all editors

In this Study, we focused on the German language version of Wikipedia and analyzed the proportion of Austrian editors as a function of article category. Altogether, we analyzed the origin of *N* = 45,491 editors who had contributed to *n* = 27 articles from the universal topic category (e.g., “Gravity”), *n* = 26 articles from the international topic category (e.g., “United Nations”), and *n* = 29 articles from the national topic category (i.e., regarding the Austrian nation, e.g., “Vienna”, see https://osf.io/sqan3/ for the full list of articles). Articles were edited on average by 554.76 editors (Range: 4 – 2395). Our preregistered hypothesis was that the percentage of Austrian editors is highest in the national article category, significantly lower in the international article category and lowest in the universal article category (https://aspredicted.org/yi4ai.pdf).

**Figure 1:**
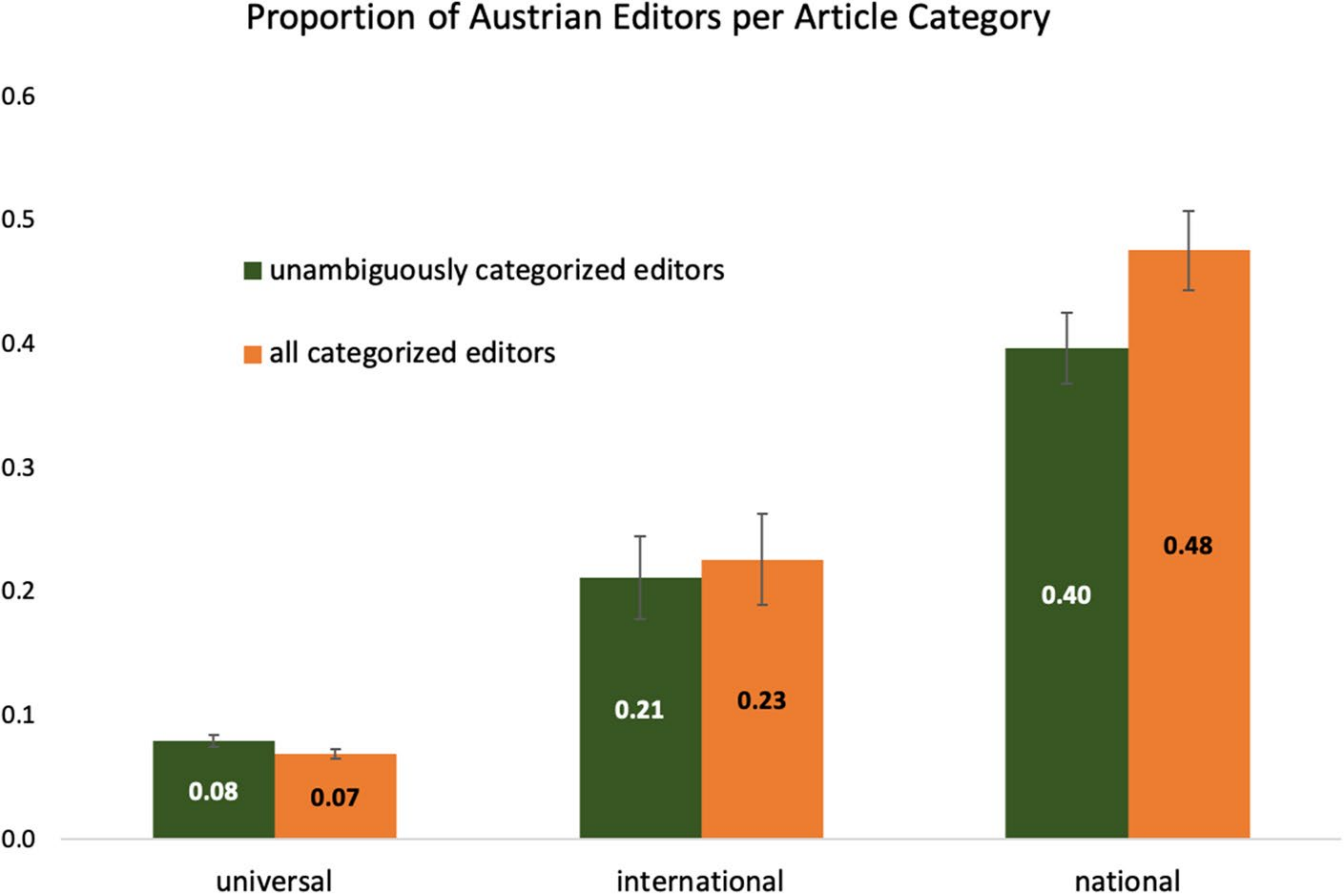
Proportion of Austrian editors as a function of article category (Sample 1).

For *N* = 33,592 editors, we could extract information regarding their nationality, and, thus determine whether they were Austrians or not. Not all of this information was perfectly reliable, however (see methods). Only for a subsample of *n* =12,008 editors, the information on user pages allowed for an unambiguous categorization. However, regardless of whether we (a) limited our analysis to those editors who could be unambiguously categorized or (b) also allowed for some uncertainty in the categorization by including also categorizations based on ambiguous information (all categorized editors), the results supported the hypothesis. The proportion of Austrians varied significantly as a function of article topic, *F*_*a*_(2, 79) = 39.523, *p* < 0.001, η_p_^2^ = 0.500; *F*_*b*_(2, 79) = 53.601, *p* < 0.001, η_p_^2^ = 0.576. The proportion of Austrians was highest for articles on national topics and distinctively so, both for unambiguously categorized editors (*M* = 0.397, *SD* = 0.154) as well as for all categorized editors (*M* = 0.476, *SD* = 0.173), as can be seen in Figure 1. It differed significantly from both universal (*M*_*a*_ = 0.080, *SD*_*a*_ = 0.025; *M*_*b*_ = 0.070, *SD*_*b*_ = 0.020), Bonferroni-corrected *p*s < 0.001, and international article topics (*M*_*a*_ = 0.212, *SD*_*a*_ = 0.172; *M*_*b*_ = 0.226, *SD*_*b*_ = 0.189), Bonferroni-corrected *p*s < 0.001. This effect could also be found when comparing universal with international topics as the international category had significantly higher proportions of Austrians than the universal category, regardless of the information basis (unambiguously categorized / all categorized editors), *p*s < .003.

Interestingly, the proportion of Austrians in the universal article category nicely matched the *overall* proportion of Austrian editors contributing to the German language version, as suggested by a previous statistic (7%, https://stats.wikimedia.org/wikimedia/squids/SquidReportPageEditsPerLanguageBreakdown.htm). This suggests that the universal article category represents the baseline and that Austrians are disproportionately represented among the contributors to international and national articles. In other words, Austrians disproportionately self-select to article topics that concern their own country.

### Sample 2—seven language versions, most prolific editors only

In order to test whether the results pattern also holds for other language versions of Wikipedia, we analyzed a set of 567 articles across seven different language versions. For each language version, we focused on one nationality of interest (e.g., Australian editors in the English language version; Canadian editors in the French language version) and defined the articles concerning the international and national topics accordingly (see methods; *n* = 189 articles from universal topics, *n* = 175 articles from international topics and *n* = 203 from national topics). From a total of *N* = 10,021 top editors across all articles we were able to extract information regarding nationality for *n* = 6,216 editors (including also ambiguous information) and to unambiguously categorize *n* = 3,705 editors. In order to test our hypothesis across language versions, the main dependent variable was the proportion of editors being of the nationality of interest per language version. Again, we compared this proportion between article categories, expecting the lowest proportion for universal articles and the highest proportion for national articles.

**Figure 2:**
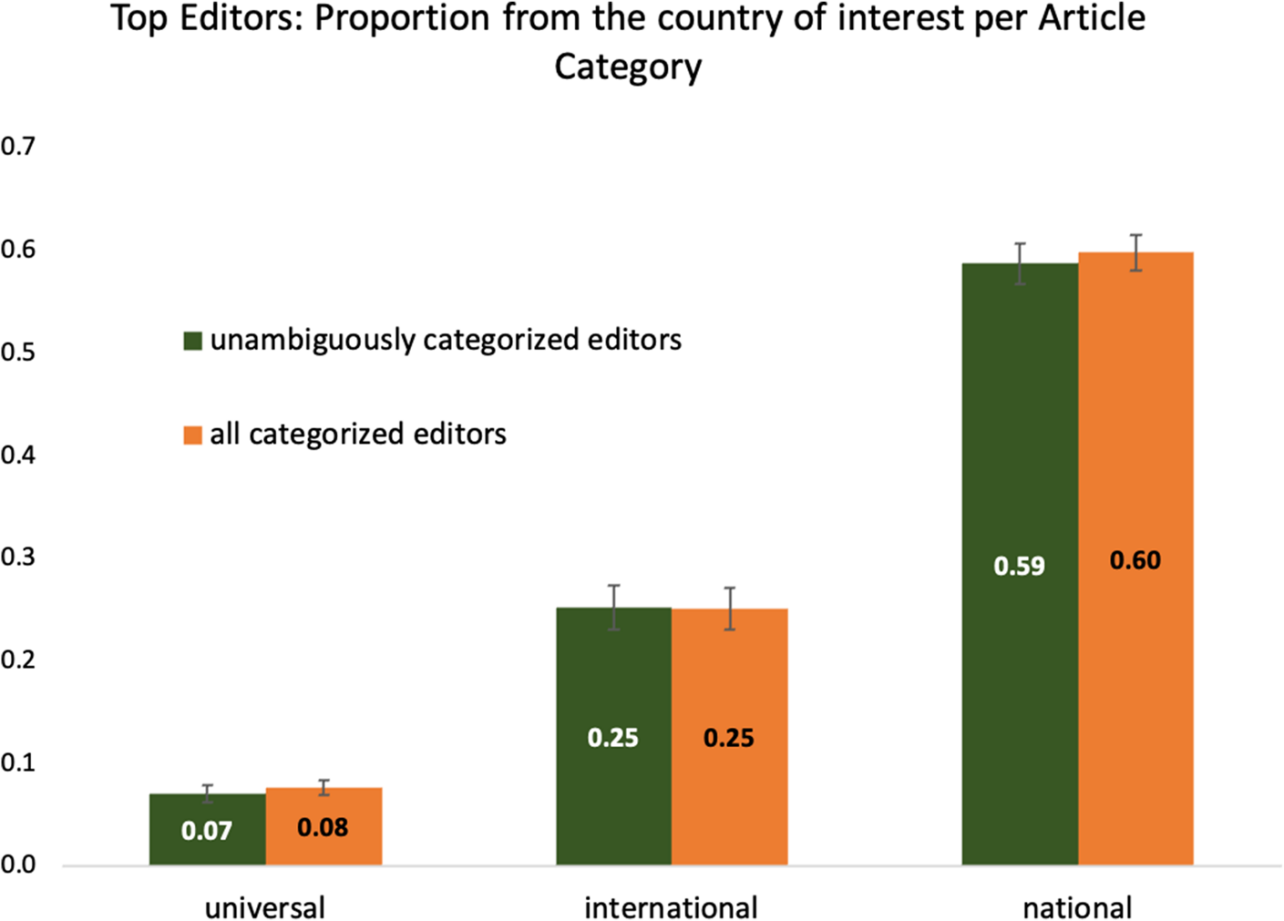
Proportion of editors from the country of interest as a function of article category for Sample 2.

Regardless of whether we (a) limited our analysis to the unambiguously categorized editors or (b) analyzed all categorized editors, the results supported the hypothesis. The proportion of members from the country of interest varied significantly as a function of article category, *F*_*a*_(2, 558) = 238.461, *p* < 0.001, η^2^ = 0.461; *F*_*b*_(2, 564) = 287.810, *p* < 0.001, η^2^ = 0.505. As can be seen in Figure 2, the proportion of editors from the country of interest was highest for articles on national topics and distinctively so (*M*_*a*_ = 0.587, *SD*_*a*_ = 0.280; *M*_*b*_ = 0.597, *SD*_*b*_ = 0.248). It differed significantly from both universal (*M*_*a*_ = 0.070, *SD*_*a*_ = 0.115; *M*_*b*_ = 0.077, *SD*_*b*_ = 0.098), Bonferroni-corrected *p*s < 0.001, and international article topics (*M*_*a*_ = 0.252, *SD*_*a*_ = 0.279; *M*_*b*_ = 0.251, *SD*_*b*_ = 0.272), Bonferroni-corrected *p*s < 0.001. This difference could also be found when comparing universal with international article topics as the international article category had a significantly higher proportion of editors from the country of interest than the universal category, regardless of whether the analysis was limited to categorizations based on certain or all information, *p*s < 0.001. Further explorations into the respective language versions yielded the same significant pattern of results (national > international > universal) with only two exceptions, where the proportion of members from the country of interest did not differ significantly between international and universal articles. The national article category, however, was distinct in all cases with the highest proportion of editors from the country of interest (see Supplemental_Material for the fully reported results).

### Sample 3—seven language versions, anonymous editors only

In order to test, whether the results pattern hinges upon manual coding, and, thus, potential bias therein, we analyzed in a third sample unregistered editors only and determined their origin solely by geo-tracking their IP-addresses. As IP-addresses only provide information about the geographic location of the connected device (if not used via a virtual private network), the data about the nationality of editors is subject to uncertainty and the sample lacks a subset of cases that could be unambiguously categorized. Across seven different language versions, 522 articles could be included into the analyses as the other ones did not comprise anonymous editors (*n* = 189 articles from universal topics, *n* = 149 articles from international topics and *n* = 184 from national topics). Altogether, we geo-tracked *N* = 178,947 editors.

**Figure 3:**
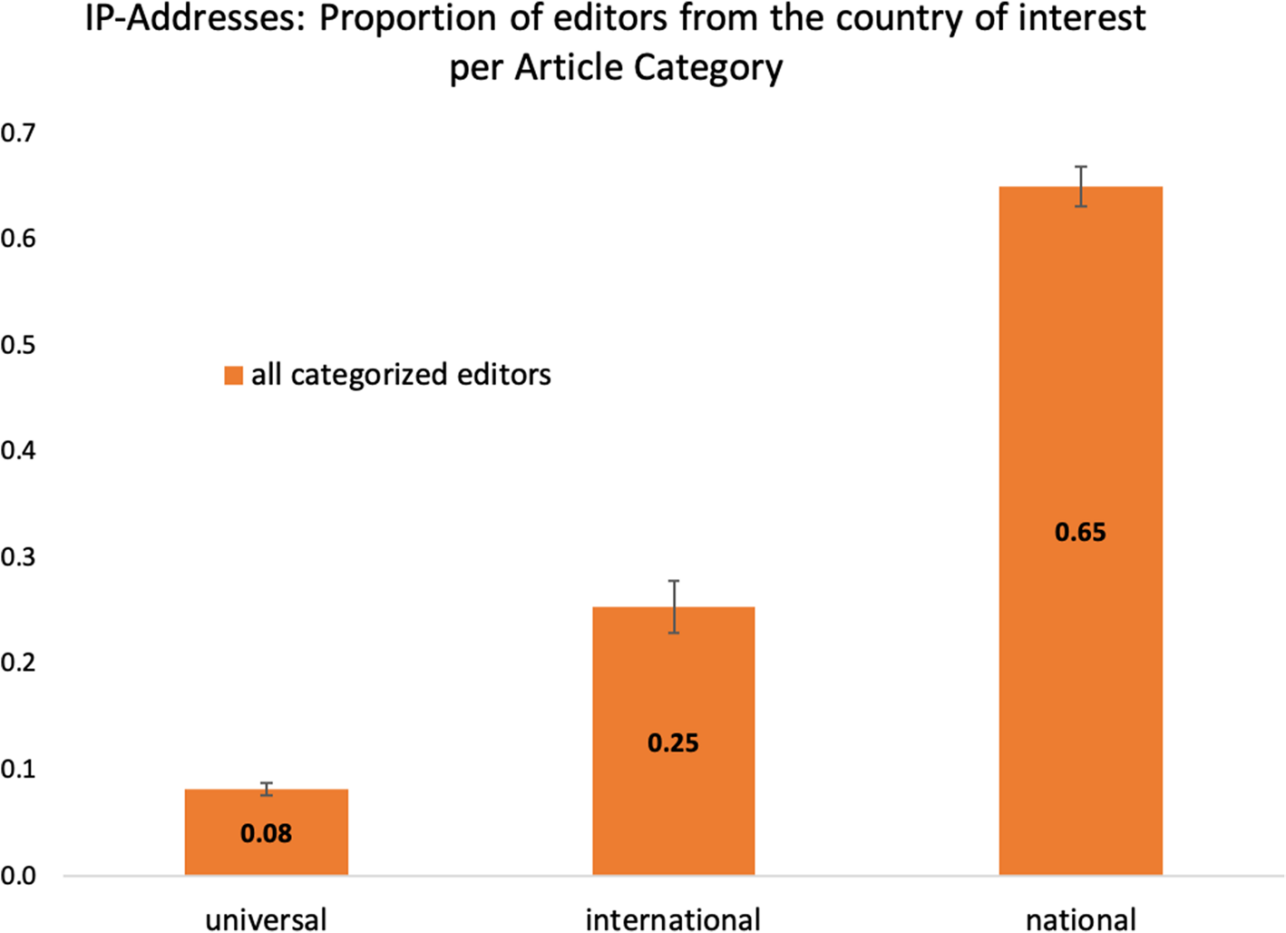
Proportion of editors from the country of interest as a function of article category for Sample 3.

The same pattern of results as in Sample 1 and Sample 2 was obtained when analyzing IP-addresses only (see Figure 3): Again, the proportion of editors from the country of interest varied significantly as a function of article category, *F*(2, 519) = 311.818, *p* < 0.001, η^2^ = 0.546. Again, the proportion of editors from the country of interest was highest for national article topics, *M* = 0.650, *SD* = 0.254, and significantly different from international article topics, *M* = 0.254, *SD* = 0.297, *p* < 0.001, as well as universal article topics, *M* = 0.082, *SD* = 0.082, *p* < 0.001. The latter two differed significantly from one another as well, *p* < 0.001. Further explorations into the respective language versions yielded the same significant pattern of results (national > international > universal) with only three exceptions, where the proportion of members from the country of interest did not differ significantly between international and universal articles. The national article category, however, was distinct in all cases with the highest proportion of editors from the country of interest (see Supplemental_Material for further information).

## Discussion

Across several language versions, hundreds of different articles, and hundreds of thousands of editors, we have documented a large self-selection effect within Wikipedia: The more exclusively an article topic is linked to a particular country, the higher the proportion of editors from that country is among the contributors (national article topics > international article topics > universal article topics). In other words, article topics of national concern (e.g., about the Austrian capital Vienna) disproportionately attract editors of that country (i.e., Austrians). The pattern of results was highly robust and reliably obtained independent of (a) the specific sample, (b) the information base (unambiguously categorized editors only vs. all categorized editors), and (c) the type of analysis (human coding vs. geo-tracking). Also, the pattern of results was nearly identical in all language versions and, thus, across different countries of interest. Only in some cases was the proportion of editors from the country of interest similar in the international and universal article category. The national article category, however, was distinct in every single instance and always entailed the largest proportion of editors from the country of interest.

When comparing our data with the *general* proportion of editors from the respective country of interest (i.e., across all articles of a language version) it becomes clear, that it is the international but even more so the national article categories that stand out: Whereas the results for the universal article category oftentimes match the proportion of editors for all articles of that language version, the proportion of editors from the country of interest exceed in the national and international article category exceed this general proportion always by a multiple. For instance, Australian editors comprise roughly 4% of the editors of the English language version of Wikipedia (https://stats.wikimedia.org/wikimedia/squids/SquidReportPageEditsPerLanguageBreakdown.htm) and their proportion among editors of the universal articles in our Sample 2 and 3 was almost identical (4–6%). Their proportion among editors of international (32–37%) and national articles (61–76%), however, was drastically increased. In our theoretical introduction, we have outlined several possible reasons for such an effect, such as ethnocentrism, increased attention to national topics due to heightened interest for but also a greater relevance to people of that nation, greater elaboration on national topics in formal education but also the news and, thus, also an easier access to information see^9^. Unfortunately, the present research may not shed light on the contributing factors. Rather, it is the first to document geographical self-selection effects within language versions of Wikipedia. It needs further research to tackle the underlying processes.

Admittedly, it might not be very surprising that articles related to a certain country are predominantly authored by editors from that country. However, please note that we did not investigate any niche topics but, instead, included articles about quite popular topics. For instance, for the national article category, we had always included the very article about the respective country, but also about its capital, head of state, famous people and geographical sites. Hence, the articles about “Austria”, “Vienna” or “Joseph Haydn”, for instance, could be expected to likewise attract editors from other countries, who have a personal connection to the country or city, who are touristic fans of geographical sites or are into music. This is even more likely in consideration of the fact that we had excluded the countries with the largest general proportion of editors in that language version. And, in fact, in the case of Austria (Sample 1), both aspects seem remarkable: first, that the proportion of Austrian editors for the national article category is about six times the proportion of the universal article category, and second, that the proportion of Austrian editors for the national article category is still – on average – below 50%. That is, even our articles from the national article category were *not* predominantly written by Austrians. Due to the fact that we analyzed popular national topics, however, one would expect even more pronounced self-selection effects for niche topics about rather unknown, local entities.

But now, why should we care about this? As outlined in the introduction, self-selection tends to result in biases. Even though we did not analyze article contents here, but only editor composition, previous research on Wikipedia has shown that editor composition matters: Editors tend to contribute information that *they* regard as relevant and accurate, which, is, however, not universally shared, and, therefore, results in a *self-focus*^62–66^. Furthermore, editors tend to contribute information that puts their own group in a systematically more positive light (*ingroup bias*^9,67^) and the higher the proportion of editors from a certain nationality, the more responsibility for an international conflict is assigned to the *other* conflicting party (*ultimate attribution error*^9,68^). There is also tentative evidence that famous people^69^ and even terrorists^70^ from a country might be presented more positively in the Wikipedia language version of that country when compared to other language versions. Translated to the article categories of the present paper, there is, thus, a risk of biased articles about national and international topics. And this risk likely increases with an increased proportion of editors coming from the respective country^9^. Consequently, the risk of biased contents is highest for articles from the national category that relate to a country that is already overrepresented among editors from that language version: Recall that the German language version is predominantly edited by Germans (> 80%, see above). As our data suggests, this general proportion equals the proportion of editors for the universal article category (see above). Consequently, one would expect even higher percentages of German editors for articles about topics that are exclusively linked to Germany (national article category) and topics that are linked to Germany as well as other countries (international article category). Thus, one would expect the overwhelming majority of editors of articles about Germany to be Germans (vs. the roughly 50% of Austrians among the editors of the national articles). Possibly, the proportion might even approach the 100% in some cases of articles. Consequently, editors of the article would be very homogeneous in at least one regard – they would have a shared nationality and may, thus, also share group-based biases^5,9,67,68^. These editor biases, in turn, may translate into biased articles as correcting alternative perspectives may be lacking^16–24^.

To be clear, however, this is speculative as we did not analyze article biases in this paper (note, that a serious analysis of article bias is very effortful, especially if it involves different languages^5,9,67^). Consequently, it is up to future research to provide more direct evidence on the link between self-selection, homogeneity among authors, and biases. Our elaborations are based, however, on well-documented biases among homogeneous collaborators (see above). Hence, article bias resulting from skewed editorship as demonstrated in our paper is certainly not inevitable, but quite likely. In order to meet its own requirements to present recognized world knowledge from a neutral point of view, (https://en.wikipedia.org/wiki/Wikipedia:No_original_research and https://en.wikipedia.org/wiki/Wikipedia:Neutral_point_of_view, retrieved on April 24, 2023.) Wikipedia should, thus, strive for more diversity among its editors – not only in general see also^71^, but also per article. But, of course, Wikipedia *does* already strive for more diversity^72^. It is difficult to accomplish, however. Not only but also because there is self-selection at every level – to editing Wikipedia^5^ as well as the specific language version and articles. For article biases that could result from the national self-selection documented in this paper, however, there might also be a workaround: As there are different language versions of Wikipedia, editors could deliberately compare how the same topic is represented in different language versions. Automatic translation tools only facilitate this possibility. And without advocating a uniform representation across language versions, such comparisons might point to systematic differences or biases (e.g., in the representation of famous people^69^), which could, then be countered – particularly, if editors were aware of the risk of bias. The present paper aimed to raise this awareness.

### Supplementary Information

Supplementary Information.

## Data Availability

All data is accessible here https://osf.io/sqan3/?view_only=dceb30cfe60d4dd7bedce69a13bc18ce.
