# Supplementary material for: How article category in Wikipedia determines the heterogeneity of its editors
Source: Sci Rep. 2024 Jan 7;14:740. doi: 10.1038/s41598-023-50448-y (PMC10772120; doi:10.1038/s41598-023-50448-y)
Supplement: Supplementary file 1 — Supplementary Information. [file 41598_2023_50448_MOESM1_ESM.docx]

**How article category in Wikipedia determines the heterogeneity of its editors**

# Supplemental Results

**Sample 1 – Inter-rater agreement**

As categorization did not only entail the judgment of whether or not a particular editor was (1) from the country of interest and/or (2) from another country than the one of interest, but could also entail the judgment that (3) no information was found for that particular editor, we analyzed inter-rater agreement once for these three categories as well as for the first two categories only. Also, we analyzed inter-rater agreement for the categorization for certain as well as the categorization based on all information. Table 1_S summarizes the findings for all analyses.

|  | Three Categories  (country of interest, other country, no information)  *N* = 1573 | Two Categories  (country of interest, other country)  *N* = 681 |
| --- | --- | --- |
| Unambiguous Categorization | Kappa = .842, *p* < .001 | Kappa = .993, *p* < .001 |
| All categorizations | Kappa = .808, *p* < .001 | Kappa = .977, *p* < .001 |

**Sample 2 – Seven Language versions, most prolific editors only**

In an exploratory attempt to analyze interacting effects of article category and language version on ingroup member proportion we used two-factor ANOVAs, which resulted in consistent effects, regardless of whether we (a) limited our analysis to the unambiguously categorized editors or (b) analyzed all categorized editors: They did not only yield the reported main effect of article category, *F_a_*(2,540) = 285.245, *p* < .001, η_p_^2^ = .514, *F_b_*(6, 540) = 353.684, *p* < .001, η_p_^2^ = .564, but also yielded a significant main effect of language version on proportion of editors from the nation of interest, *F_a_*(6, 540) = 16.438, *p* < .001, η_p_^2^ = .154, *F_b_*(6, 540) = 18.735, *p* < .001, η_p_^2^ = .171, as well as a significant interaction between article category and language version, *F_a_*(12, 540) = 3.238, *p* < .001, η_p_^2^ = .067, *F_b_*(12, 540) = 3.267, *p* < .001, η_p_^2^ = .067.

In order to elucidate the interactions and to test, whether the predicted pattern (national > international > universal) holds in every language version, we ran additional univariate ANOVAs separate for every language version with only article category as between-article factor. Below, we report descriptive statistics per language version and denote significant differences between article categories by different indices per line (i.e., different superscripted letters *per line* indicate significant differences, whereas identical superscripted letters *per line* denote no significant differences between the respective article categories, see OSF for the full statistics).

1. **Unambiguously categorized editors only**

|  |  | article category | | |
| --- | --- | --- | --- | --- |
|  |  | national | international | universal |
| language version of Wikipedia | German | .687^a^  *(.270)* | .239^b^  *(.239)* | .087^c^  *(.113)* |
|  | English | .759^a^  *(.261)* | .328^b^  *(.293)* | .050^c^  *(.091)* |
|  | French | .446^a^  *(.227)* | .167^b^  *(.286)* | .065^b^  *(.098)* |
|  | Dutch | .653^a^  *(.296)* | .361^b^  *(.311)* | .137^c^  *(.191)* |
|  | Portuguese | .707^a^  *(.191)* | .452^b^  *(.287)* | .113^c^  *(.105)* |
|  | Russian | .404^a^  *(.218)* | .190^b^  *(.175)* | .043^c^  *(.070)* |
|  | Spanish | .445^a^  *(.260)* | .079^b^  *(.211)* | .000^b^  *(.000)* |

Table 1_S: Mean proportion of editors of the nation of interest per article category for each language version (standard deviations in parentheses)

Note: different superscripted letters per line indicate significant differences between article categories

1. **All categorized editors**

|  |  | article category | | |
| --- | --- | --- | --- | --- |
|  |  | national | international | universal |
| language version of Wikipedia | German | .648^a^  *(.248)* | .240^b^  *(.265)* | .118^b^  *(.135)* |
|  | English | .764^a^  *(.248)* | .355^b^  *(.298)* | .037^c^  *(.064)* |
|  | French | .435^a^  *(.233)* | .151^b^  *(.230)* | .069^b^  *(.070)* |
|  | Dutch | .634^a^  *(.190)* | .355^b^  *(.279)* | .094^c^  *(.105)* |
|  | Portuguese | .778^a^  *(.143)* | .384^b^  *(.268)* | .161^c^  *(.100)* |
|  | Russian | .491^a^  *(.176)* | .190^b^  *(.193)* | .043^c^  *(.063)* |
|  | Spanish | .421^a^  *(.201)* | .113^b^  *(.258)* | .015^b^  *(.036)* |

Table 2_S: Mean proportion of editors of the nation of interest per article category for each language version (standard deviations in parentheses)

Note: different superscripted letters per line indicate significant differences between article categories

**Sample 3 – seven language versions, anonymous editors only**

In an exploratory attempt to analyze interacting effects of article category and language version on ingroup member proportion we used two-factor ANOVA, which resulted in a significant main effect of language version on proportion of ingroup members as well as a significant interaction between article category and language version, *F_a_* (6, 501) = 18.168, *p* < .001, η_p_^2^ = .179; *F_a_* (12, 501) = 4.210, *p* < .001, η_p_^2^ = .092.

1. **All categorized editors**

|  |  | article category | | |
| --- | --- | --- | --- | --- |
|  |  | national | international | universal |
| language version of Wikipedia | Arabic | .634^a^  *(.229)* | .109^b^  *(.265)* | .006^b^  *(.013)* |
|  | English | .613^a^  *(.25)* | .378^b^  *(.342)* | .057^c^  *(.010)* |
|  | French | .504^a^  *(.313)* | .111^b^  *(.111)* | .121^b^  *(.048)* |
|  | Dutch | .770^a^  *(.143)* | .492^b^  *(.307)* | .216^c^  *(.056)* |
|  | Portuguese | .822^a^  *(.134)* | .239^b^  *(.319)* | .132^c^  *(.064)* |
|  | Russian | .702^a^  *(.164)* | .248^b^  *(.264)* | .035^c^  *(.015)* |
|  | Spanish | .487^a^  *(.294)* | .132^b^  *(.193)* | .005^b^  *(.003)* |

Table 3_S: Proportion of editors of the nation of interest per article category for each language version (standard deviations in parentheses)

Note: different superscripted letters per line indicate significant differences between article categories
